# Biallelic modification of *IL2RG* leads to severe combined immunodeficiency in pigs

**DOI:** 10.1186/s12958-016-0206-5

**Published:** 2016-11-03

**Authors:** Jung-Taek Kang, Bumrae Cho, Junghyun Ryu, Caitlin Ray, Eun-Jin Lee, Yun-Jin Yun, SunMi Ahn, JinSeok Lee, Dal-Young Ji, Nathaniel Jue, Sherrie Clark-Deener, Kiho Lee, Kwang-Wook Park

**Affiliations:** 1 MGENPLUS Biotechnology Research Institute, Seoul, 08511 South Korea; 2 Department of Animal Science & Technology, Sunchon National University, Suncheon, 57922 South Korea; 3 Department of Animal and Poultry Sciences, Virginia Tech, Blacksburg, VA USA; 4 Department of Large Animal Clinical Sciences, Virginia-Maryland College of Veterinary Medicine, Virginia Tech, Blacksburg, VA USA

**Keywords:** *IL2RG*, Severe Combined Immunodeficiency (SCID), CRISPR/Cas9, Somatic cell nuclear transfer (SCNT), Knockout pigs

## Abstract

**Background:**

Pigs with SCID can be a useful model in regenerative medicine, xenotransplantation, and cancer cell transplantation studies. Utilizing genome editing technologies such as CRISPR/Cas9 system allows us to generate genetically engineered pigs at a higher efficiency. In this study, we report generation and phenotypic characterization of *IL2RG* knockout female pigs produced through combination of CRISPR/Cas9 system and SCNT. As expected, pigs lacking IL2RG presented SCID phenotype.

**Methods:**

First, specific CRISPR/Cas9 systems targeting *IL2RG* were introduced into developing pig embryos then the embryos were transferred into surrogates. A total of six fetuses were obtained from the embryo transfer and fetal fibroblast cell lines were established. Then *IL2RG* knockout female cells carrying biallelic genetic modification were used as donor cells for SCNT, followed by embryo transfer.

**Results:**

Three live cloned female piglets carrying biallelic mutations in *IL2RG* were produced. All cloned piglets completely lacked thymus and they had a significantly reduced level of mature T, B and NK cells in their blood and spleen.

**Conclusions:**

Here, we generated *IL2RG* knockout female pigs showing phenotypic characterization of SCID. This *IL2RG* knockout female pigs will be a promising model for biomedical and translational research.

**Electronic supplementary material:**

The online version of this article (doi:10.1186/s12958-016-0206-5) contains supplementary material, which is available to authorized users.

## Background

The *IL2RG* gene, located on the X chromosome, encodes the common gamma chain protein [35], which is a subunit of various interleukin receptors that are involved in immune system. The receptor is a key part of major lymphocytes, therefore, supports the growth and maturation of several subtypes of lymphocytes: T cells, B cells, and natural killer (NK) cells. These lymphocytes are an essential component of the adaptive and innate immune system. Deletion or mutation in the *IL2RG* gene would lead to the loss of functional immune system. Specifically, *IL2RG* mutation results in X-linked severe combined immunodeficiency (XSCID), characterized by profound defects in cellular and humoral immunity in humans [2, 17, 24]. Transgenic mice lacking functional *IL2RG* exhibits SCID phenotypes due to limited number of mature B and T cells and the loss of NK cells [3]. These mice have been a useful resource for immunological, inflammation, oncology, and stem cell transplantation studies [9, 32, 36]. However, rodent models do not always recapitulate the genetic and physiological states of humans. In fact, there are significant differences in immune system between mice and humans [22]. For example, expression and ligand specificity of Toll-like receptors, which can activate innate immune are different between human and mouse [43]. Likewise, post-inflammatory genomic responses in mouse models poorly mimic human [31]. In addition, because of their size and shorter lifespan compared to humans, mouse models are not ideal to carry out surgical and clinical procedures or employ long-term tracking and evaluation of tissue or cell transplantation. Therefore, immunological assessments and procedures developed using SCID mice as a model may not translate directly into the same outcomes in humans.

Pigs are considered to be a leading large animal model in biomedical research because they share similar anatomy and physiology with humans [26]. Various pig models have been generated to study human diseases such as cystic fibrosis [28], diabetes mellitus [27], Alzheimer’s disease [13], and retinitis pigmentosa [29]. SCID pigs, in particular, can be a useful model in regenerative medicine, xenotransplantation, and cancer cell transplantation researches because of similarities in immune system between pigs and humans. Disruption of *IL2RG* in male pigs resulted in immunodeficiency presented in X-linked SCID patients [34]; these pigs lacked T and NK cells [34, 38]. Disruption of other key genes related to immune response also resulted in the production of SCID pigs lacking T and B cells [8, 14].

Conventionally significant effort is required to generate these SCID pigs due to technical limitations. However, recent advancement in genome editing technologies such as CRISPR/Cas9 system allows us to generate genetically engineered pigs at a higher efficiency and in a short period of time, less than six months [14, 16]. The CRISPR/Cas9 system, originated from a natural microbial immune system [1], consists of a RNA-guided Cas9 endonuclease, a single guide RNA (sgRNA), and the trans-activating CRISPR RNA (tracrRNA) which have been engineered for genome editing in eukaryotic cells [4]. This CRISPR/Cas9 system has emerged as an efficient and powerful tool for gene editing [11, 39], and been successfully applied in many mammals, including mice, rats, pigs, and monkeys [6, 18, 19, 23, 37].

Here, we applied CRISPR/Cas9 technology to target *IL2RG* during porcine embryogenesis, and generated *IL2RG* knockout fibroblast cells from fetuses derived from the embryos. Then the *IL2RG* knockout cells were used as nuclear donors to produce *IL2RG* knockout female pigs by somatic cell nuclear transfer (SCNT). As a result, SCID pig models lacking mature lymphocytes were generated, which could be a valuable large animal model for human disease research or biomedical study.

## Methods

### Reagents

All chemicals in the study were purchased from Sigma–Aldrich Chemical Company (St. Louis, MO, USA) unless indicated otherwise.

### Animals

All experiments involving animals were approved by the Institutional Animal Care and Use Committee of the institute of MGENPLUS co., Korea, and Virginia Tech (#14-019). All the procedures were conducted under the guidelines of the Committee. All surgical procedures were performed under general anesthesia, and all necessary efforts were made to minimize any potential suffering of animals. Pigs were maintained under conventional housing conditions.

### Design and construction of IL2RG targeting CRISPR/Cas9 system

The sgRNA that could recognize porcine *IL2RG* gene were designed using an online CRISPR design tool (http://zifit.partners.org/ZiFiT/Disclaimer.aspx). Sequence information of the designed sgRNAs is 5′-CGAAGGTCCTCACGCACAGT**GGG**-3′ (gRNA #1) and 5′-CCGAAGGTCCTCACGCACAG**TGG**-3′ (gRNA #2), respectively. The PAM can be identified by the bold font in each sgRNA. Specificity of the designed sgRNAs was confirmed by searching for similar porcine sequences in GenBank. Both sgRNAs are designed to create DSB in exon 1 of *IL2RG*. The sgRNA sequences were introduced into the px330 vector (Addgene) as described previously (Additional file 1: Table S1) [40]. Then the targeting vectors were used as a template to generate sgRNA and Cas9 mRNA through in vitro transcription (Additional file 1: Table S2).

### Generation of IL2RG knockout fetuses by direct injection of CRISPR/Cas9 system into early embryos

For in vitro maturation, cumulus oocyte complex (COC) were maturated in vitro in a TCM-199 based maturation media containing 0.5 IU/ml FSH, 0.5 IU/ml LH, 0.82 mM cysteine, 3.02 mM glucose, 0.91 mM sodium pyruvate, and 10 ng/ml EGF. After 42–44 h of maturation, cumulus cells were removed by incubating the oocytes into a media containing 0.1 % hyaluronidase. Oocytes that extruded the first polar body were used for in vitro fertilization (IVF). Then mature oocytes, groups of 25–30 oocytes, were placed in 50 μ l droplets of IVF medium (modified Tris-buffered medium with 113.1 mM NaCl, 3 mM KCl, 7.5 mM CaCl_2_, 11 mM glucose, 20 mM Tris, 2 mM caffeine, 5 mM sodium pyruvate, and 2 mg/ml BSA). Extended semen was washed with PBS three times then the sperm pellet was resuspended with mTBM media. Then, 50 μl sperm (2.5 × 10^5^ sperm/ml) was introduced into mTBM drops that contained oocytes. The gametes were co-incubated for 5 h. Presumptive fertilized embryos were then placed in Porcine Zygote Media 3 (PZM-3) [41] at 38.5 °C, 5 % CO_2_, and 5 % O_2_ incubator until microinjection of CRISPR/Cas9 system. After 2–4 h post-IVF, presumable zygotes were injected with RNA form of CRISPR/Cas9 system to target *IL2RG*. Concentrations of 10 ng/μl sgRNA and 20 ng/μl Cas9 mRNA was injected into the cytoplasm of fertilized oocytes using a FemtoJet microinjector (Eppendorf, Hamburg, Germany). Microinjection was conducted in manipulation medium (TCM199 with 0.6 mM NaHCO_3_, 2.9 mM HEPES, 30 mM NaCl, 10 ng/ml gentamicin, and 3 mg/ml BSA) on the heated stage of a Nikon inverted microscope (Nikon Corporation, Tokyo, Japan). Injected zygotes were washed then transferred and cultured into PZM-3. Embryos used for embryo transfer were cultured in PZM-3 in the presence of 10 ng/ml GM-CSF [15] until embryo transfer. A total of 245 microinjected embryos were transferred into two surrogate sows at day 5 or 6 post-IVF. The embryos were surgically transferred into the oviduct of the sows.

### Establishing fibroblast cells from IL2RG knockout fetuses

For the collection of fetal fibroblast cells, porcine fetuses were obtained on day 40 of gestation. Genomic DNAs were isolated from each fetus using PureLink Genomic DNA kit (Thermo Fisher Scientific, Waltham, MA, USA) following the manufacturer’s instructions. PCR to genotype modifications on *IL2RG* was conducted using Platinum Taq DNA Polymerase (Thermo Fisher Scientific). PCR conditions were as follows, initial denature at 95 °C for 2 min, denature at 95 °C for 30 s, annealing at 55 °C for 30 s and extension at 72 °C for 30 s for 34 cycles. The amplicons were sent to VBI (Biocomplexity institute of Virginia) for sequencing (primer information is in Additional file 1: Table S3). Using extended primers, fetus #3 and #6 were conducted PCR again. PCR conditions were as follows, initial denature at 95 °C for 2 min, denature at 95 °C for 30 s, annealing at 55 °C for 30 s and extension at 72 °C for 2 min for 34 cycles. The fetuses were cut into small pieces and digested with 0.25 % trypsin–0.02 % EDTA for 30 min at 37 °C. Following trypsinization, the cells were washed by centrifugation and subsequently seeded on to culture dishes and cultured in DMEM (Gibco BRL, Grand Island, NY, USA) supplemented with 15 % fetal bovine serum (HyClone #AVM90621, USA) and 1 % penicillin/streptomycin under 5 % CO_2_ at 37.5 °C. After 3 days of culture the tissue explants were removed by rinsing the flask with Dulbecco’s phosphate buffered saline (DPBS; Gibco BRL) and the remaining attached fibroblast cells were cultured until confluence.

### Detection of mutations on IL2RG generated by the CRISPR/Cas9 system

Genomic DNA from each cell colony was extracted using a DNA extraction kit (iNtRon Biotechnology, Seongnam-si, Korea), following the manufacturer’s instructions. To confirm genetic modifications on *IL2RG* from the cell colonies, PCR was conducted using 2× Taq. Premix (PCR Biosystems, London, UK). PCR was performed at 40 cycles with porcine *IL2RG* specific primers using the following conditions; one cycle of initial-denaturation at 95 °C for 5 min followed by 40 cycles of denaturation at 95 °C for 60 s, annealing at 52 °C for 30 s, and elongation at 72 °C for 30 s, and a cycle of post-elongation at 72 °C for 10 min. Additional file 1: Table S3 shows sequences of primers used for genotyping. Mutations on *IL2RG* gene were assessed by digesting PCR amplicons with the T7 endonuclease I (T7E1) enzyme as previously described [12]. The PCR products from the DNA isolated from colonies were denatured at 95 °C for 5 min and re-annealed at room temperature for 10 min, then digested by T7E1 (ToolGen labs, Seoul, Korea) at 37 °C for 0.5 h. Digestion of the PCR products was expected if the colony contained mutated *IL2RG*. PCR products with potential modification of *IL2RG* were confirmed by sequencing.

### Somatic cell nuclear transfer and embryo transfer

SCNT was performed as described in previous studies [12, 42]. Pig ovaries were collected from a local abattoir and transported to the laboratory in 0.9 % (w/v) NaCl solution at 25–30 °C. Oocytes were aspirated from antral follicles (3–6 mm in diameter) and cultured in maturation medium at 39 °C with 5 % CO_2_ at 100 % humidity. After 44 h of maturation, denuded oocytes which extruded the first polar body were used for SCNT. Mature MII oocytes were enucleated by aspirating the first polar body and adjacent cytoplasm with a thin glass pipette (20 um in diameter) in manipulation medium supplemented with cytochalasin B (5 mg/ml stock, 1.5 ul per 10 ml manipulation medium). Then a single donor cell was injected into the perivitelline space of enucleated oocytes. Oocyte cytoplasm-cell complexes were then fused and activated by electric pulse (ECM 2001; BTX Inc., San Diego, CA, USA, two DC pulses of 1.1 kV/cm for 60 μsec). Reconstructed embryos were cultured in PZM3 in 5 % CO_2_ at 39 °C with 0.5 uM Scriptaid, a histone deacetylase inhibitor, for 14–16 h. Embryos with an intact plasma membrane were surgically transferred into the oviduct of a surrogate (average 230 embryos) at the two day after observed estrus. Successful pregnancy was assessed by an ultrasound at day 28 days post embryo transfer. The gestation was monitored every 2 weeks. After approximately 114 days, cloned piglets were delivered by c-section from recipients. On the day of birth, a tale biopsy was performed on each piglet for genomic DNA extraction and genotyping.

### Flow Cytometric Analysis (FACS)

Peripheral blood mononuclear cells (PBMCs) and splenocytes were isolated from whole blood and spleen from *IL2RG* knockout pigs and age-matched control pigs. To identify CD3^+^, CD4^+^, and CD8^+^ T cells and CD21^+^ B cells, mouse anti-pig CD3e (Southern Biotech, AL, USA), CD4a, CD8a, and mouse anti-human CD21 (BD Pharmingen, CA, USA) were used in this study. Mouse anti-pig CD16 (AbD Serotec, NC, USA) and mouse anti-pig monocyte and granulocyte (M/G, BD Pharmingen, CA, USA) were also used in this study for detection of NK cell population. A total of 5 × 10^5^ PBMCs or splenocytes were incubated with the indicated Abs for 40 min at 4 °C and washed twice with PBE. At least 10,000 cells were analyzed per run. Samples were analyzed using a FACS Calibur system with CELLQUEST software (BD Bioseciences, CA, USA). Each experiment was repeated at least three times.

### Histological analysis

Spleens from *IL2RG* knockout and age-matched wild-type pigs were first fixed in 10 % neutral buffered formalin. The fixed tissues were embedded in paraffin and sectioned for H&E staining and immunohistochemistry (IHC). In IHC, rabbit anti-pig CD3 antibody (abcam, MA, USA) as T lymphocytes marker and mouse anti-pig CD79a antibody (abcam, MA, USA) as B lymphocytes marker were used for analysis of distribution of T and B lymphocytes.

## Results

### Design of CRISPR/Cas9 vector and isolation of IL2RG knockout cells

**Fig. 1:**
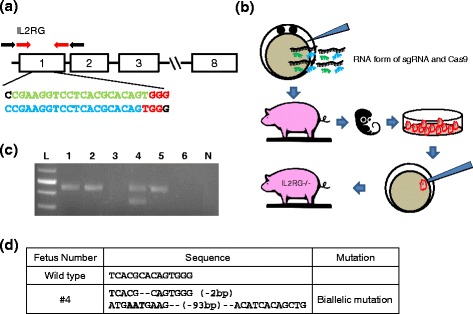
Use of CRISPR/Cas9 system to disrupt *IL2RG* in pigs. **a** Design of CRISPRs to target *IL2RG*. Sequences in green and blue indicate target sequences and letters in red reflect PAM (NGG) sequences. Red arrows indicate the location of primers used to genotype embryos and piglets. Black arrows indicate the location of extended primers. **b** Schematic strategy used to produce IL2RG deficient pigs. *IL2RG* knockout cells lines were established from fetuses derived from CRISPR/Cas9 injected embryos. SCNT was performed to generate *IL2RG* knockout female pigs. **c** PCR amplification to genotype *IL2RG* knockout fetuses. Various mutations were detected from fetus 1, 2, 4, and 5. **d** Genotype of IL2RG knockout cell line used for SCNT 4. Bold letters indicate insertion or change in nucleotides, and ‘-’ indicates deletion of nucleotide

Porcine *IL2RG* is located on the X chromosome and consists of 8 exons [7]. In this study, we constructed CRISPR/Cas9 systems that can target exon 1 of porcine *IL2RG*, which contains the first translation initiation site (Fig. 1a). Schematic construction of the CRISPR/Cas9 system used in this study is shown in Fig. 1b. When 10 ng/μl sgRNA and 20 ng/μl Cas9 mRNA were introduced into developing embryos and subsequent embryos were genotyped, all embryos carried mutations in *IL2RG*; no wild-type sequence was found from the in vitro analysis (Table 1). Transferring IVF embryos injected with the RNA form of CRISPR/Cas9 system resulted in one pregnancy (Table 2) and six fetuses were collected from the surrogate to establish *IL2RG* knockout cell lines. The fetuses and cell lines were screened by the T7E1 assay and PCR DNA sequencing to detect potential mutation generated by the CRISPR/Cas9 system. Mutations in the *IL2RG* were found in fetus #1, #2, #4, and #*5*. No PCR amplicon was acquired from fetuses #3 and #6 even by using extended primers, suggesting that two fetus carried large deletions (>2 kb) by CRISPR/Cas9 system (Fig. 1c). The efficacy of introducing mutations in the *IL2RG* was 100 % (Additional file 1: Table S4). Genotyping fetus #4 indicated that the cells carried biallelic mutation (2 bp or 93 bp deletion) in *IL2RG*. The cells were used to generate *IL2RG* knockout female pigs (Fig. 1d). The results demonstrate that the CRISPR/Cas9-mediated targeting is effective in generating mutations in the genome of porcine developing embryos.

**Table 1:** Efficacy of CRISPR/Cas9 system to induce targeted disruption of IL2RG during embryogenesis in vitro. CRISPR/Cas9 system at the concentration of 10 ng/ul each sgRNAs and 20 ng/ul Cas9 mRNA was introduced into pig zygotes. Genotyping of subsequent embryos on day 7 demonstrated that all embryos carried mutation on *IL2RG*; no wild type sequence was found from genotyping

Concentration of CRISPR/Cas9 (ng/uL)	# of embryos injected	% of blastocyst on day 7 (number of blastocysts/cleaved)	# of blastocyst genotyped	Genotypes				
				Homozygous Mutation	Biallelic Mutation	Mosaic Mutation	Heterozygous Mutation	Wild-type
10/20	70	25.71 % (18/70)	4	0	2	2	0	0

**Table 2:** Embryo transfer results. A total of five embryo transfers were performed in this study. Transferring CRISPR/Cas9 injected IVF embryos was to generate *IL2RG* knockout fetuses. *IL2RG* knockout piglets were produced by transferring SCNT embryos

Surrogate ID	Number of embryos generated	Number of embryos transferred into a recipient	Source of embryos	pregnancy	Number of Fetus/piglets
73-10	128	124	IVF	No	–
Y463	117	93	IVF	Yes	6
104–154	262	262	SCNT	Yes	3
28–35	283	283	SCNT	No	–
27–78	352	352	SCNT	No	–

### Production of IL2RG knockout pigs

**Fig. 2:**
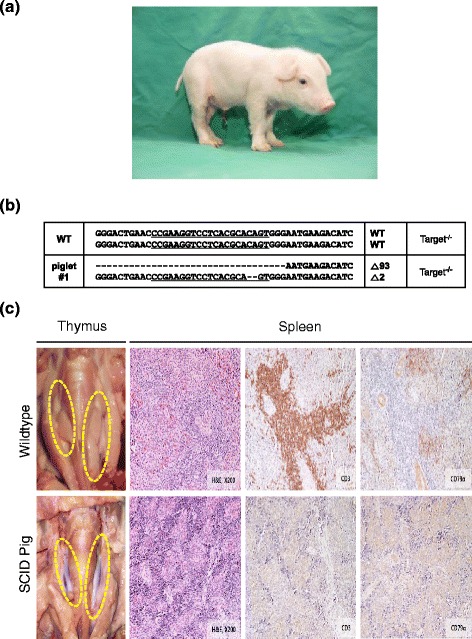
Generation of *IL2RG* knockout pigs. **a** An image of *IL2RG* knockout pigs produced in this study (**b**) Genotypes of *IL2RG* knockout female piglets. The dots indicate the deletion mutation in the sequence of *IL2RG* knockout pigs. **c** Immunodeficiency presented in *IL2RG* knockout pigs. The thymic phenotype in *IL2RG* knockout pigs shows absence of thymus compared to normal thymuses in wild-type pigs. Dotted yellow circle indicates the location of thymus. The histological analysis of the spleens of wild-type and *IL2RG* knockout pigs indicate that *IL2RG* knockout pigs lack T and B cells (Original magnification 200×)

*IL2RG* knockout pigs were produced by SCNT from the targeted fibroblast cells. Reconstructed embryos were transferred to three surrogates, and one of the surrogates had a full-term pregnancy (Table 2). Three female cloned pigs were obtained from the recipient via cesarean section (Fig. 2a). PCR genotyping, T7E1 assay and DNA sequence analyses of the 3 cloned pigs showed that all 3 pigs had the same mutation as the nuclear donor cells (2 bp and 93 bp deletion, Fig. 2b). Three survivors died from pneumonia and severe arthritis or unknown cause(s) (all were euthanized) between postnatal day 1 (P1) and P12.

### Phenotypic characterization of IL2RG knockout pigs

All piglets were raised under standard housing conditions and all the *IL2RG* knockout piglets presented health issues. Necropsy results from the knockout pigs and age-matched wild-type demonstrated that the *IL2RG* piglets were immunodeficient. The biallelic *IL2RG* knockout piglets lacked thymus compared with age-matched wild-type pigs (Fig. 2c). Although there was no obvious difference in size, the spleens of biallelic *IL2RG* knockout pigs were much thinner and more loosely packed than those of age-matched wild-type pigs. The spleens were fixed and embedded for H&E staining to further analyze its inner structure and cell composition. The spleens of biallelic *IL2RG* knockout pigs were hypoplastic in the central artery of periarterial lymphatic sheath, and lacked white pulp (Fig. 2c). In IHC, CD3 positive T lymphocytes and CD79α positive B lymphocytes were absent or less existed in collected spleen samples of all piglets, showing that the number of lymphocytes was remarkably reduced in the biallelic *IL2RG* knockout pigs compared with the wild-type controls (Fig. 2c). However, one littermate having longest survival period (d12) have some CD79α positive B lymphocytes alike other littermate.

**Fig. 3:**
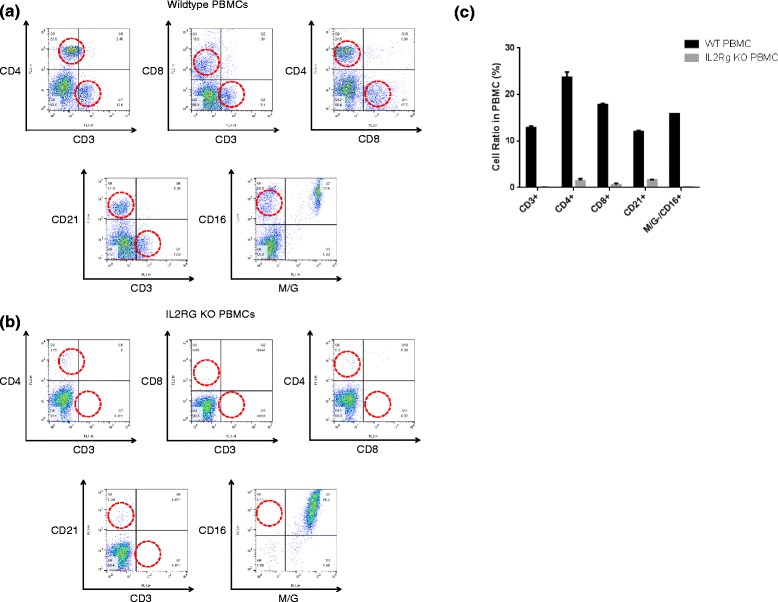
Flowcytometric analysis of *IL2RG* knockout pigs. **a** Flowcytometric analysis of T, B and NK cells in the peripheral blood mononuclear cells and splenocytes of wild-type piglets. **b** Flowcytometric analysis of T, B and NK cells in the peripheral blood mononuclear cells and splenocytes of *IL2RG* knockout pigs. In figure (A) and (B), the dot plots show CD3^+^, CD4^+^ and CD8^+^ cells for the demarcation of T cell subpopulations and CD3^−^, CD21^+^ cells for the differentiation of B cell, M/G^−^ cells and CD16^+^ cells for the differentiation of NK cell subpopulations. **c** The proportion ratio of T, B and NK cells in PBMC of wild-type and *IL2RG* knockout pigs

To detect the status of B, T lymphocytes and NK cells in the biallelic knockout pigs, cells were collected from spleen and whole blood. Then the cells were used to perform FACS assay. Staining the splenocytes and PBMC with CD3, CD4 and CD8 Abs demonstrated that the biallelic knockout pigs had almost no CD4/CD8 single- and double-positive (+) cells compared with the wild-type controls. The ratio of CD3^+^CD4^+^ and CD3^+^CD8^+^ cells also decreased drastically in the knockout pigs, whereas ratios of about 20 % (respectively) were observed in the wild-type counterparts, indicating the lack of T lymphocytes in PB and spleen. The average number of B cell (CD21^+^) was significantly lower in the knockout piglets compared with the wild-type. However, B cells were detected from a cloned piglet with the longest survival period (P12); the level was 36.7 %, similar to 36.5 % in spleen of wildtype, compared with other littermate lacking B cells (4.86 %). This result is consistent with IHC result. The number of NK cells (M/G^−^, CD16^+^) was notably lower in *IL2RG* knockout pigs than wild-type pigs (Fig. 3).

## Discussion

*IL2RG* is responsible for growth and maturation of immune cells such as T cells and NK cells because it is a common component of many interleukin receptors. In this study, we generated *IL2RG* knockout pigs by using CRISPR/Cas9 system-mediated gene targeting strategy. These knockout pigs presented SCID phenotype as expected. Because of the SCID phenotype, the *IL2RG* knockout pigs can be used as research model for in vivo stem cell repopulation. *IL2RG* homozygous knockout mouse model has been an excellent recipient model for engraftment of human cells [10]. For instance, *IL2RG* null mice have significantly improved engraftment results compared with other immunocompromised SCID model when human cord blood engraftment was attempted [21]. The *IL2RG* knockout pig model can be a more useful animal model considering the discrepancy between immune cell function and the immune system of humans and rodents.

In this study, we injected CRISPR/Cas9 systems directly into developing embryos to target *IL2RG*. The efficacy of this approach was effective as all resulting embryos and fetuses carried mutation in *IL2RG*. Because the approach may lead to animals with different genotypes and mosaicism [20], fetal fibroblast cells from each fetus were genotyped and used for SCNT to generate *IL2RG* knockout pigs. With this approach, exact genotype of nuclear donor cells can be identified prior to the production of cloned animals; this can assure the production of animals only carrying desired modification. Using this approach, *IL2RG* knockout pigs were produced at a higher rate compared to traditional gene targeting approach which utilizes endogenous homologous recombination mechanism in somatic cells. Because *IL2RG* is located on the X chromosome and generating animals carrying multi-allelic modifications is challenging, previous reports of *IL2RG* knockout pigs were all in male [38]. However, using the CRISPR/Cas9 system, we could generate female *IL2RG* deficient pigs. A recent study demonstrates generation of *RAG2/IL2RG* knockout females [16], however, to our best knowledge, this is the first report of female IL2RG deficient pigs.

Opportunistic infections in SCID animals after birth are unavoidable under conventional housing conditions. We therefore recovered full-term *IL2RG* knockout piglets recovered via cesarean section (114 d of gestation) to avoid any risk of infection during parturition. However, the *IL2RG* knockout pigs could not thrive and only lasted a short period (<12 days) because of unavoidable opportunistic infection due to their SCID condition under conventional housing conditions, not pathogen-free facilities..At postmortem examination, these animals have showed a pleural fluid inside the thoracic cavity, supporting evidence of infection (data not shown). The early death of the *IL2RG* knockout pigs is probably because of deficient in functional immune system. Generally, long-term maintenance of severely immunodeficient animals would require housing under pathogen-free conditions. Therefore, management of additional *IL2RG* animals should be conducted at facilities severely controlled against exogenous pathogens.

A marked decrease in the number of T and B cells has been reported in XSCID mice [3, 5] and rat [20]. In human XSCID patients, although the number of T and NK cells is significantly decreased, the number of B cells remains normal or is occasionally increased [2, 33]. Similarly, *IL2RG* knockout pigs produced in previous studies lacked T and NK cells but showed normal B cell populations, and identical phenotypic characteristics were shown identically in human XSCID [34, 38]. However, some *IL2RG* knockout pigs obtained in this study showed an absent or lower B cell population; the level of T and NK cells was lower as expected, although one littermate have similar B cell population with control. This discrepancy could come from gender biased effect. As mentioned above, all the previous reports of *IL2RG* modifications in pigs were in males. And most of human XSCID cases are also in male. Interestingly, some reports in mice indicate that there is difference in immune responses of SCID mice based on the gender. Female SCID mice were more effective in supporting engraftment of foreign cells compared to their male counterpart [21, 25]. Specifically, repopulation experiment of human hematopoietic stem cells using female immunodeficient mice (NOD/SCID/*IL2RG* -null) showed that female recipients displayed higher engraftment efficiency compared to male [25]. In this study, the difference in B cell population among cloned littermates carrying same genetic modification was unexpected. We speculate that this discrepancy is probably due to unexpected changes in epigenetic make-up of the X chromosome. Mammalian *IL2RG* orthologs are typically located on the X chromosome and in female one of the X chromosomes is inactivated during early development. Clones are known to have abnormally skewed pattern of X inactivation [30], and this could be a reason behind the differences in the level of B cells among *IL2RG* knockout pigs produced in this study. Our further studies will focus on the functional differences in *IL2RG* between genders in pigs. Also, modification of epigenetic pattern in same littermate produced in this study will be additionally studied.

## Conclusions

Genome editing by CRISPR/Cas9-mediated technology represents a practical strategy for the production of genetically engineered pigs. Using this technology enabled inactivation of *IL2RG* gene in pigs. In this study, we generated *IL2RG* knockout female pigs showing phenotypic characterization of SCID. This *IL2RG* knockout female pig model will greatly contribute not only to cancer and stem cell research but also to preclinical evaluations of the transplantation of pluripotent stem cells, such as iPS cells.

## Additional file

Additional file 1: Table S1. Oligos used to introduce sgRNA into px330. Each pair of forward and reverse primers were annealing and ligated into the PX330 vector. **Table S2.** Primers used to generate template DNA for in vitro transcription to produce sgRNA and mRNA form of Cas9. **Table S3.** Primers used to genotype *IL2RG* mutations introduced by CRISPR/Cas9 system. The extend primers were used to genotype *IL2RG* from fetus 3 and 6. **Table S4.** The mutation of fetus. Two fetus contained hemizygous mutation in IL2RG, other two fetus had biallelic mutation, and 2 fetus had presumable large deletion (>1.9kb). (DOCX 22 kb)

## Abbreviations

COC: Cumulus oocyte complex
DPBS: Dulbecco’s phosphate buffered saline
IHC: Immunohistochemistry
NK: Natural killer
PBMCs: Peripheral blood mononuclear cells
PZM-3: Porcine zygote media 3
SCNT: Somatic cell nuclear transfer
sgRNA: Single guide RNA
T7E1: T7 endonuclease I
tracrRNA: Trans-activating CRISPR RNA
XSCID: X-linked severe combined immunodeficiency
