# Supplementary material for: Biallelic modification of IL2RG leads to severe combined immunodeficiency in pigs
Source: Reprod Biol Endocrinol. 2016 Nov 3;14:74. doi: 10.1186/s12958-016-0206-5 (PMC5095964; doi:10.1186/s12958-016-0206-5)
Supplement: Additional file 1: Table S1. — Oligos used to introduce sgRNA into px330. Each pair of forward and reverse primers were annealing and ligated into the PX330 vector. Table S2. Primers used to generate template DNA for in vitro transcription to produce sgRNA and mRNA form of Cas9. Table S3. Primers used to genotype IL2RG mutations introduced by CRISPR/Cas9 system. The extend primers were used to genotype IL2RG from fetus 3 and 6. Table S4. The mutation of fetus. Two fetus contained hemizygous mutation in IL2RG, other two fetus had biallelic mutation, and 2 fetus had presumable large deletion (>1.9kb). (DOCX 22 kb) [file 12958_2016_206_MOESM1_ESM.docx]

Additional file 1

Table S1. Oligos used to introduce sgRNA into px330. Each pair of forward and reverse primers were annealing and ligated into the PX330 vector.

| Oligo | Sequence (5’- 3’) |
| --- | --- |
| IL2RG F1 | CAC CCG AAG GTC CTC ACG CAC AGT |
| IL2RG R1 | AAA CAC TGT GCG TGA GGA CCT TCG |
| IL2RG F2 | CAC CCC GAA GGT CCT CAC GCA CAG |
| IL2RG R2 | AAA CCT GTG CGT GAG GAC CTT CGG |

Table S2. Primers used to generate template DNA for in vitro transcription to produce sgRNA and mRNA form of Cas9.

| Primer | Sequence (5’- 3’) |
| --- | --- |
| T7 IL2RG F1 | TTA ATA CGA CTC ACT ATA GGC GAA GGT CCT CAC GCA CAG T |
| T7 IL2RG F2 | TTA ATA CGA CTC ACT ATA GGC CGA AGG TCC TCA CGC ACA G |
| T7 sgRNA R1 | AAA AGC ACC GAC TCG GTG CC |
| Cas9 F | TAA TAC GAC TCA CTA TAG GGA GAA TGG ACT ATA AGG ACC ACG AC |
| Cas9 R | GCG AGC TCT AGG AAT TCT TAC |

Table S3. Primers used to genotype *IL2RG* mutations introduced by CRISPR/Cas9 system. The extend primers were used to genotype *IL2RG* from fetus 3 and 6.

| Primer | Forward primer (5’- 3’) | Product size |
| --- | --- | --- |
| IL2RG F1 | CAG AGG ATT TAG CCT GTG TCA TAG CAT ACA TTG | 425bp |
| IL2RG R1 | CCC AGT ACT CTA AAA TTT TGC CCA CAT CCT TC |  |
| IL2RG F1 extend | CGG TAA TAA TCA TGA CTA GAG GGA ATG AAA GAT TGA TTT ATC | 1904bp |
| IL2RG R1 extend | GAT CCC TCA CTT CTT CTT TTC TGC CCA AGT AC |  |

Table S4. The mutation of fetus. Two fetus contained hemizygous mutation in IL2RG, other two fetus had biallelic mutation, and 2 fetus had presumable large deletion (>1.9kb)

| Fetus | sex | Mutation of IL2RG |
| --- | --- | --- |
| 1 | Male | Hemizygous mutation |
| 2 | Male | Hemizygous mutation |
| 3 | Male | Not amplified |
| 4 | Female | Biallelic mutation |
| 5 | Female | Biallelic mutation |
| 6 | Male | Not amplified |
